# A Fatal Case of Inversio Partialis Uteri Puerperalis*Read before the Chicago Gynæcological Society, Nov 29, 1878.

**Published:** 1879-01

**Authors:** Edward Warren Sawyer

**Affiliations:** Lecturer on Obstetrics and Diseases of Children, Rush Medical College, Chicago


### ARTICLE II.

### A FATAL CASE OF INVERSIO PARTIALIS UTERI PUERPERALIS.*

By EDWARD WARREN SAWYER, Lecturer on Obstetrics and Diseases of Children, Rush Medical College, Chicago.

Mrs. B——, 38 Aldine square, æt. 35 years, of small, frail physique; multipara, last labor five years ago. She fell in labor at midnight, Dec. 19, 1877; an hour afterward, I saw her for the first time. I was struck by the unusual size of the abdomen, which suggested multiple pregnancy. The lower extremities

* Read before the Chicago Gynæcological Society, Nov 29, 1878.

were very œdematous, and had been in this condition for several months.

Upon examination, I found the os two-thirds opened, the vertex in advance, and still very high. Her pains, which returned with natural regularity and frequency, were short and sharp, lacking in force; they were not well borne by the patient, who frequently demanded ether.

At 2 a. m. the os was fully opened. Upon rupturing the membranes, there escaped the greatest quantity of liquor amnii I have ever encountered, the water inundating the bed and falling upon the floor. Her abdomen soon assumed the size usual to this moment. The head was well engaged in the excavation, in the third position of the vertex (R. O. P.), the anterior fontanelle occupying the centre of the field. In auscultating the tumor, I heard but one fœtal heart, on the median line just below the umbilicus; pulsations 144 in the minute. After waiting for several contractions of the uterus, which appreciably advanced the head, I began to allow the patient to inhale ether during the accession of each pain. During the intervals, she was quite conscious, and kept up an intelligible conversation with her husband and her attendant. The effect of this obstetrical degree of anæsthesia was most happy; the woman bore her pains without complaint, and they were noticeably increased in force, though still shorter and weaker than natural.

At 4:30 a. m. the occiput had rotated forward, into the second position, and the head was well engaged in the outlet of the canal. I now carried the anæsthesia to the surgical degree for a moment, applied my short forceps, and delivered the head within ten minutes. All ether was discontinued from this moment. There was a pause of at least five minutes, when I became solicitous for the infant, who did not close the gums upon my finger in the mouth, and so completed the extraction by hooking my finger into the left axilla. During this time, the husband, under my direction, was compressing the uterus with both hands; this compression he continued until relieved by me. The child required a vigorous slapping and cold douche before it would breathe.

After the lapse of about ten minutes—the woman in the meantime seemed in a natural sleep—I passed my finger into the vagina, and found the placenta partly extruded through the mouth of the uterus ; I completed its delivery with less difficulty than usually obtains. The patient wakened at this moment, and simply said, "Don't." I examined the abdomen, and felt the uterus very flaccid and reaching to the umbilicus, *but uniformly rounded.*

A few minutes later, not more than fifteen — the husband in the meantime keeping up compression—I noticed a pallor of the woman's face, who opened her eyes and yawned. I quickly raised the bed-covering, and saw that a rather brisk hæmorrhage was going on ; the pulse was rapid and very feeble. I put my hand upon the belly, but *could not plainly feel the outlines of the uterus.* I sunk my hand deep downward, and had but little difficulty in compressing the aorta against the vertebral column, though the woman complained that my pressure was uncomfortable ; this procedure completely arrested the hæmorrhage. As soon as the nurse could pour a teaspoonful of Squibb's fluid extract of ergot from a bottle, it was given to her. I called for ice, which was not in the house. While the husband went for ice, I slapped the abdomen with a cold wet towel. The dose of ergot was repeated twice in the next half hour.

Ice was soon procured, and I hastened to rub the epigastrium with a lump ; I also passed a lemon-shaped piece into the vagina as far as the mouth of the uterus, which presented nothing unusual. The uterus immediately contracted, and its outlines were easily made out through the abdominal parietes ; the upper border was not now rounded upward, as the fundus usually is, but was represented by a straight, hardened edge, extending transversely across the hypogastrium, at a point nearly midway between the symphysis pubis and umbilicus. This edge was quite like the rim of a bowl. At either side, and resting upon this rim, a spherical body, continuous with a rounded cord, could be easily felt (the ovary and tube).

It now flashed upon me that the fundus of the uterus had fallen downward into the cavity of the organ. This discovery was made about forty minutes after the delivery of the placenta, and some twenty-five minutes after I had once felt the fundus flaccid, but rounded upward. I ceased my compression of the aorta, and no more blood was lost. From this moment, the uterus was in a state of tonic spasm, *as if completely ergotized.* At no time could I detect the slightest relaxation, though I longingly watched for this event.

I passed my hand into the vagina, and found the uterus unusually high; the os was opened to the extent of about four centimeters, and rigid. A segment of the prolapsed fundus could be felt looking through, and in contact with the open mouth. The contrast in the feel of this circular area of spongy fundus, with the smooth covering of the vaginal portion of the cervix, made it of easy recognition.

I shaped my hand into a cone, which I impinged against the fundus, through the os, at the same time making a *point d'appui* with my left hand upon the upper rim of the uterus, through the abdominal walls. In this manner I was enabled to exercise a degree of force in attempting to push the fundus upward, such as I feared would tear the uterine tissue; but I made no impression upon it; the organ felt like cartilage. The patient fainted during this effort at replacement; the pulse disappeared from the wrist, and I was obliged to desist. A dose of morphia, with brandy, was given by the mouth, and brandy was also thrown into the rectum. The trunk was surrounded with bottles of hot water, and frictions faithfully used; the foot of the bed was also raised. In a half hour, these measures were rewarded by a marked improvement; the color had returned to her face, and the pulse was quite strong at the wrist. I now made a seeond attempt at replacement, but with the same futile result; the poor woman complained of pain, and again fainted. The respiration soon became sighing; there was great jactitation, and our united efforts were required to keep her upon the bed. She was delirious, jumped from one side of the bed to the other, crying, "I shall be uncovered," and no efforts of ours could prevent her kicking herself naked. The *ensemble* constituted a scene which can never be effaced from memory.

All efforts at resuscitation were kept up faithfully, but without avail. The unfortunate lady soon became moribund, and died very quietly at eight o'clock, about three hours after delivery, rather more than two hours after the inversion was recognized, and about one hour after the second effort at replacement of the fundus.

Dr. Byford reached the house some ten minutes before dissolution, and recognized the utter hopelessness of the case.

The abdomen was examined fifty-five hours after death, and the uterus, which was preserved, was found in the state already described. Its tissue was not torn. There was no difficulty in replacing the fallen fundus at this time.

The child was a male, and weighed ten pounds.

### REMARKS.

This accident, which is one of the gravest that can befall a puerperal woman is, fortunately, one of extreme rarity; since it occurred but "once in upwards of 190,800 deliveries at the Rotunda Hospital." *

The result in this case was not exceptional.† Of seven cases seen by Hamilton, "in one single instance only the woman survived the shocking accident." ‡

It is a common observation, that the loss of blood and shock are greater in the incompletely inverted uterus, when the fundus is grasped by the body, than when the organ is turned quite inside out. § The most common variety is the incomplete ||; hence the great mortality attending the accident.

The reflections suggested by the case in point are: first, as to the cause of the accident. The undue distension of the uterus by the liquor amnii undoubtedly weakened the uterine walls, so that, when the organ was emptied, the fundus could easily fall into the cavity of the body. This enfeeblement was recognized before delivery, through the short, sharp, weak pains. The almost universal observation made by the older authors, that inversion of the uterus is generally caused by the attendant dragging upon the cord of the undetached placenta, has absolutely no application here; for the placenta was entirely detached spontaneously. Second, as to the management of the accident.

---

* Playfair, Treatise of Midwifery, Philadel., 1876.

† Velpeau, Traité de l'art des Accouhemens, Paris, 1835.

‡ Hamilton, Outlines of Midwifery, Edinburgh, 1784.

§ Kilian, Die Operative Geburtshülfe, Bonn, 1835.

|| Mauriceau, Traitié des Maladies des Femmes Grosses, Paris, 1740.

Writers agree upon the importance of immediate replacement of the inverted organ. Surely it is to the reposition of the organ that we are to look for a relief from dangerous shock, and security against hæmorrhage. As a rule, according to authors, the immediate replacement of the fundus is not difficult. But an insurmountable obstacle in the way of such success in this case was the condition of tonic spasm which at first seemed due to the administration of ergot. One cannot refrain from expressing a regret at this point, though it is true that the effects of ergot were indicated by the post-partum atony of the uterus. There is consolation in the fact, moreover, that some portion of the uterus is usually spasmodically contracted, when there is atony of the organ, when no ergot has been given. § ||

This patient succumbed to the shock incident to having the fundus grasped by the spasmodically contracted body of the uterus. The shock may have been augmented, under the circumstances, by the loss of blood; but I have more than once seen a greater hæmorrhage with no appreciable effects upon the woman.

§ Dewees, System of Midwifery, Phil., 1824.

|| Smith, Lectures on Obstetrics, Gardner, New York, 1858.

